# Bis-indolic compounds as potential new therapeutic alternatives for tularaemia

**DOI:** 10.3389/fcimb.2014.00024

**Published:** 2014-02-27

**Authors:** Yvan Caspar, Vivien Sutera, Sandrine Boisset, Jean-Noël Denis, Max Maurin

**Affiliations:** ^1^Laboratoire de Bactériologie, Centre Hospitalier Universitaire de Grenoble, Université Joseph Fourier Grenoble-1Grenoble, France; ^2^Laboratoire Adaptation et Pathogénie des Micro-organismes, UMR-5163 CNRS, Université Joseph Fourier Grenoble-1Grenoble, France; ^3^Département de Chimie Moléculaire (SeRCO), UMR-5250 CNRS, Université Joseph Fourier Grenoble-1Grenoble, France

**Keywords:** tularaemia, *Francisella tularensis*, bis-indolic compounds, antibacterial activity

## Abstract

*Francisella tularensis* is the etiological agent of tularaemia and a CDC class A biological threat agent. Few antibiotic classes are currently useful in treating tularaemia, including the aminoglycosides gentamicin and streptomycin, fluoroquinolones, and tetracyclines. However, treatment failures and relapses remain frequent and *F. tularensis* strains resistant to antibiotics have been easily selected *in vitro*. In this study, we evaluated the activity of new synthetic bis-indole derivatives against this pathogen. Minimum inhibitory concentrations (MICs) of four compounds (dcm01 to dcm04) were determined for the reference strains *F. tularensis* subsp. *holarctica* LVS NCTC10857, *F. tularensis* subsp. *novicida* CIP56.12 and *F. philomiragia* ATCC25015, and for 41 clinical strains of *F. tularensis* subsp. *holarctica* isolated in France. Minimal bactericidal concentrations (MBCs) were determined for the dcm02 and dcm04 compounds for the LVS and two clinical strains. Killing curves were also determined for the same three strains exposed to dcm04. All tested bis-indole compounds were bacteriostatic against *F. tularensis* subsp. *holarctica* strains, with a MIC_90_ of 8 μg/mL for dcm01, dcm02, and dcm03, and 2 μg/mL for dcm04. Only one strain was resistant to both dcm01 and dcm03, with MICs > 32 μg/mL. In contrast, *F. tularensis* subsp. *novicida* was resistant to all derivatives and *F. philomiragia* was only susceptible to dcm02 and dcm04, with MICs of 16 and 4 μg/mL, respectively. MBC and killing curve experiments revealed significant bactericidal activity (i.e., 3-log reduction of the bacterial inoculum) of the dcm02 and dcm04 compounds only for the LVS strain. In conclusion, we have identified novel synthetic bis-indole compounds that are active against *F. tularensis* subsp. *holarctica*. They may be drug candidates for the development of new therapeutic alternatives for tularaemia treatment. Their further characterization is needed, especially identification of their bacterial targets.

## Introduction

*Francisella tularensis*, the agent of the zoonosis tularaemia, may cause severe to fatal human infections. This intracellular, Gram-negative bacterium is highly infectious for humans and many animal species. No human-to-human transmission has been described so far and human infection may occur through direct contact with infected animals, ingestion of contaminated meat or water, arthropod bites, contact with contaminated environments and laboratory exposure to *F. tularensis* cultures (Dennis et al., 2001; Maurin et al., 2011). *F. tularensis* is a class A biological threat agent according to the CDC (Centers for Diseases Control and Prevention, Atlanta, Georgia, USA). The highly virulent *F. tularensis* subsp. *tularensis* strains (Jellison type A) are located in North America, whereas *F. tularensis* subsp. *holarctica* strains (Jellison type B) are found throughout the northern hemisphere. In Europe, tularaemia cases are often sporadic (Maurin et al., 2011), but outbreaks have recently been reported in many countries, including in Spain, Norway and Sweden (Pérez-Castrillón et al., 2001; Larssen et al., 2011; Rydén et al., 2012). The first-line therapy of tularaemia is based on a reduced number of antibiotics, including the aminoglycosides (gentamicin and streptomycin), the tetracyclines (e.g., doxycycline), and the fluoroquinolones (e.g., ciprofloxacin) (Johansson et al., 2002). Treatment duration is usually 7–10 days for gentamicin and ciprofloxacin, and 2–3 weeks for doxycycline. However, high rates of failure and relapse are observed in tularaemia patients, especially when treatment is delayed and/or lymph node suppuration occurs (Rotem et al., 2012).

The aminoglycosides such as gentamicin and streptomycin have a bactericidal activity against *F. tularensis in vitro*, and their use in tularaemia patients is associated with almost 100% cure rates (Kaya et al., 2011; Rotem et al., 2012). However, they are nephro- and ototoxic and can only be administrated parenterally (Tärnvik and Chu, 2007). Gentamicin is currently used in many countries where streptomycin is no longer available. However, treatment failures with this antibiotic have recently been reported in 11 paediatric patients with oropharyngeal tularaemia in Turkey, with successful recovery after switching to streptomycin (Kaya et al., 2011). Doxycycline can be administrated orally, with few side effects. However, the tetracyclines are contraindicated in children under 8 years of age and in pregnant women because of the risk of permanent staining of the dental enamel and bone toxicity in the foetus (Tärnvik and Chu, 2007; Kaya et al., 2011). Treatment with this bacteriostatic antibiotic is associated with higher relapse rates as compared to aminoglycosides and fluoroquinolones, especially when treatment is delayed and/or of short duration (Dennis et al., 2001; Tärnvik and Chu, 2007). Doxycycline is administered for a minimum of 14 days (Dennis et al., 2001; Tärnvik and Chu, 2007). The fluoroquinolones (especially ciprofloxacin and levofloxacin) are preferred as first-line drugs for treatment of tularaemia cases of mild to moderate severity (Johansson et al., 2002). They are bactericidal against *F. tularensis in vitro*, orally administrable, and have few side effects. They can be administrated to young children but not to pregnant women (Johansson et al., 2000; Dennis et al., 2001; Tärnvik and Chu, 2007; Kaya et al., 2011). Ciprofloxacin is recommended as first-line drug in case *F. tularensis* is used as a biological weapon (Dennis et al., 2001; Rotem et al., 2012).

Other antibiotics such as the beta-lactams, the macrolides, cotrimoxazole, chloramphenicol, and rifampicin are not recommended for treatment of tularaemia. Beta-lactams are not effective both because they are inactivated by the class A beta-lactamase produced by *F. tularensis* (Antunes et al., 2012) and they are poorly effective against the intracellular form of this pathogen (Maurin et al., 2000). The macrolides are considered unreliable for treatment of tularaemia because most *F. tularensis* strains have natural high-level resistance to these antibiotics. Only azithromycin may be a possible alternative in pregnant women infected with type B biovar I strains (Dentan et al., 2013). Chloramphenicol and cotrimoxazole are poorly effective *in vitro* and potentially associated with severe side effects (Tärnvik and Chu, 2007). Rifampicin is active against *F. tularensis in vitro*, but its use as a monotherapy is usually associated with rapid selection of resistant mutants.

No natural strains of *F. tularensis* with acquired resistance to gentamicin, fluoroquinolones or doxycycline have been isolated so far. However, *in vitro* experiments have shown that mutants resistant to fluoroquinolones, rifampicin or macrolides can be selected easily (Tärnvik and Chu, 2007; Gestin et al., 2010; Sutera et al., 2014). At present, treatment failures and relapses are considered to be primarily related to delayed administration of appropriate antibiotic therapy rather than *in vivo* selection of antibiotic-resistant mutants (Dennis et al., 2001; Johansson et al., 2002; Kaya et al., 2011; Rotem et al., 2012). However, the bioengineering of genetically modified strains of *F. tularensis* resistant to first-line drugs for use as a biological warfare agent is a major concern. Thus, innovative antibiotics with original structures and bacterial targets, active against this highly virulent pathogen, would be beneficial not only to improve treatment efficacy in tularaemia patients, but also to reinforce our preparedness against the misuse of antibiotic-resistant *F. tularensis* strains.

We recently identified synthetic bis-indole derivatives as new antistaphylococcal compounds with preserved activity against multi-drug resistant strains of *Staphylococcus aureus*, including MRSA strains (Denis et al., 2013a,b). In this study, we evaluated the activity of four of the leading compounds against clinical isolates of *F. tularensis* subsp. *holarctica*.

## Materials and methods

### Bis-indolic compounds and antibiotics

**Figure 1:**
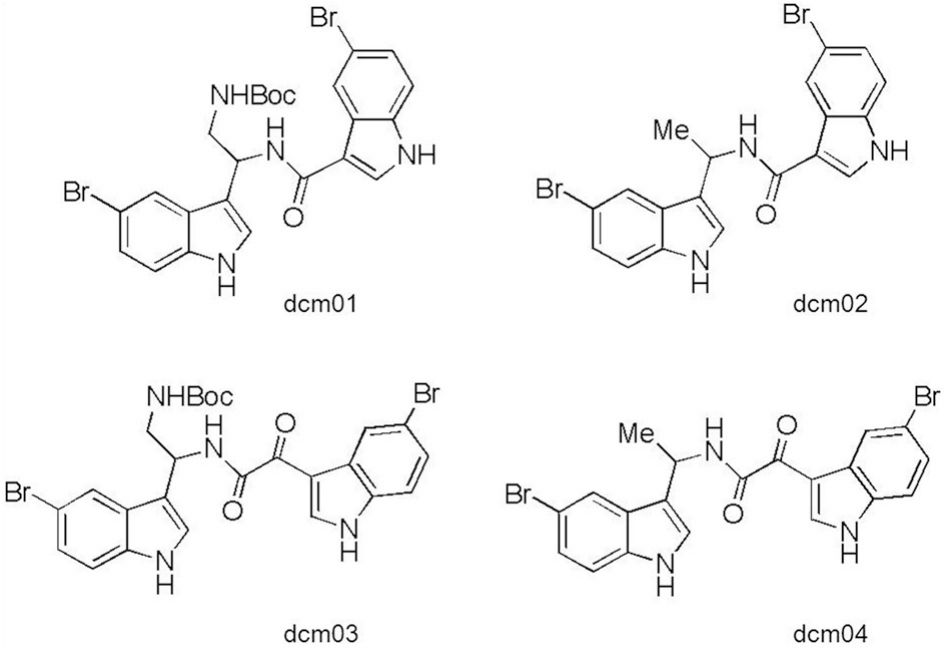
**Structures of the four bis-indole compounds**.

The four bis-indolic compounds evaluated in this study (dcm01, dcm02, dcm03, and dcm04) were synthesized by the DCM (Département de Chimie Moléculaire, Université Joseph Fourier Grenoble-1, Grenoble, France) according to previously published protocols (Denis et al., 2013a,b). The structures of the tested bis-indole compounds are presented on Figure 1. We also used gentamicin (Panpharma, Fougères, France) and doxycycline (Sigma-Aldrich, Lyon, France) as controls. Stock solutions of the bis-indolic compounds were prepared at 12.8 g/L in 100% DMSO (Sigma-Aldrich, Lyon, France) and stock solutions of gentamicin and doxycycline were prepared in sterile distilled water. All were kept frozen at −80°C until used.

### Bacterial strains

All experiments were conducted in a biosafety level 3 laboratory. The use of *F. tularensis* strains was carried out under the approval of ANSM (Agence nationale de sécurité du médicament et des produits de santé). We tested 41 isolates (Ft1–Ft41) of *F. tularensis* subsp. *holarctica* identified to the subspecies level in our laboratory (French reference center for *Francisella*) by amplification and sequencing of the intergenic 16S-23S rRNA region (Maurin et al., 2011). Four isolates were obtained from dead hares and the 37 others from human samples (Table 1). The clinical strains corresponded to independent and sporadic tularaemia cases occurring throughout France between 2004 and 2013 (Maurin et al., 2011). We also tested reference strains including *F. tularensis* subsp. *holarctica* LVS NCTC10857, *F. tularensis* subsp. *novicida* CIP56.12 and *F. philomiragia* ATCC25015. *S. aureus* ATCC29213 was tested as a control strain susceptible to the tested bis-indole compounds (Denis et al., 2013a,b). The reference bacterial strains were purchased from the American Type Culture Collection (ATCC, Mannasas, VA, USA) or the Collection of the Pasteur Institute (CIP, Centre de Ressource Biologique de l'Institut Pasteur, Paris, France). All strains are kept frozen in cryotubes (MastDiagnostic, Amiens, France) at −80°C. When needed, they are grown on chocolate agar supplemented with Polyvitex® (CHA-PVX medium, bioMérieux, Marcy l'Etoile, France) at 37°C in a 5% CO_2_-enriched atmosphere.

**Table 1:** **Sources of the 41 isolates of *F. tularensis* subsp. *holarctica* (Ft1 to Ft41) used in this study**.

**Strain**	**Host**	**Year of isolation**	**Clinical sample**
Ft1	Animal	UNK	Hare tissue
Ft2	Animal	UNK	Hare tissue
Ft3	Animal	UNK	Hare tissue
Ft4	Animal	UNK	Hare tissue
Ft5	Human	2004	Blood culture
Ft6	Human	2007	Blood culture
Ft7	Human	2006	Conjunctivitis
Ft8	Human	2007	Cutaneous ulcer
Ft9	Human	2007	Blood culture
Ft10	Human	2008	Mediastinal lymph node
Ft11	Human	2008	Pharynx
Ft12	Human	2008	Pharynx
Ft13	Human	2008	Pharynx
Ft14	Human	2008	Blood culture
Ft15	Human	2008	Cerebrospinal fluid
Ft16	Human	2008	UNK
Ft17	Human	2008	UNK
Ft18	Human	2008	Blood culture
Ft19	Human	2008	Blood culture
Ft20	Human	2008	Skin ulcer
Ft21	Human	2008	Conjunctivitis
Ft22	Human	2009	Whitlow
Ft23	Human	2009	Middle ear
Ft24	Human	2009	Lymph node
Ft25	Human	2010	Blood culture
Ft26	Human	2010	Blood culture
Ft27	Human	2010	Blood culture
Ft28	Human	2010	Lymph node
Ft29	Human	2010	Blood culture
Ft30	Human	2011	Lymph node
Ft31	Human	2011	UNK
Ft32	Human	2012	UNK
Ft33	Human	2011	Blood culture
Ft34	Human	2012	Blood culture
Ft35	Human	2012	Finger abscess
Ft36	Human	2012	UNK
Ft37	Human	2010	Blood culture
Ft38	Human	2012	Blood culture
Ft39	Human	2012	Whitlow
Ft40	Human	2012	Pleural fluid
Ft41	Human	2013	Pleural fluid

*UNK, unknown*.

### Determination of the minimum inhibitory concentrations

Minimum inhibitory concentrations (MICs) of the four bis-indolic compounds were determined against *S. aureus* ATCC29213, *F. philomiragia* ATCC25015 and all *F. tularensis* strains, using a broth microdilution method recommended by the Clinical and Laboratory Standards Institute (CLSI; M07-A8 Vol. 29, No. 2). Mueller-Hinton 2 broth supplemented with 2% PolyViteX® (MH2-PVX, bioMérieux, Marcy L'Etoile, France) was used as the antibiotic susceptibility testing medium for *F. tularensis* strains because of their fastidious nature. MH2 alone was used for other species. One row of a 96-well microtiter plate was filled with 75 μL of twofold serial dilutions of the tested bis-indolic compound in MH2-PVX medium, so as to obtain final bis-indolic concentrations ranging from 0.06 to 32 μg/mL in 0.5% DMSO. A bacterial inoculum (75 μL per well, 5 × 10^5^ CFU/mL of final inoculum) was then added to each well. Antibiotic free cultures containing 0.5% DMSO were used as DMSO toxicity controls. MH2-PVX medium with 0.5% DMSO served as a negative control. Microplates were incubated at 37°C in a 5% CO_2_ atmosphere. The MICs were read after 18 h culture incubation for *S. aureus* ATCC29213 and 48 h for *F. tularensis* strains. MICs corresponded to the minimum bis-indolic compound concentration that allowed complete inhibition of visual growth of bacteria. Experiments were conducted at least twice to confirm results. Following the same procedure but without DMSO, the MICs of gentamicin and doxycycline were determined against *F. tularensis* subsp. *holarctica* Ft6 and Ft24 strains and the control strain *S. aureus* ATCC29213.

### Determination of the minimum bactericidal concentrations

Minimum bactericidal concentrations (MBCs) were determined in triplicate experiments following CLSI recommendations (CLSI, M26-A, Vol. 19, No. 18), for the two most active compounds (dcm02 and dcm04) against three *F. tularensis* subsp. *holarctica* strains: the LVS strain and the two clinical strains Ft6 and Ft24. We used the same microdilution broth method described for MIC determination, but the primary bacterial inoculum was 10^6^ CFU/mL. MBCs of gentamicin and doxycycline were determined in parallel as a bactericidal and a bacteriostatic control, respectively. After 48 h incubation at 37°C in a 5% CO_2_ atmosphere, MBCs were determined by plating 50 μL of tenfold serial dilutions of the bacterial suspensions of wells with no visible growth, and of the antibiotic-free control well, onto CHA-PVX medium. CFU counts were determined after 72 h incubation of the plates at 37°C, in a 5% CO_2_ atmosphere. The detection limit was 20 CFU/mL. The MBC corresponded to the minimal antibiotic concentration which resulted in at least 99.9% reduction of the primary bacterial inoculum (i.e., 3 log_10_ reduction of bacterial titers).

### Time-kill curves

Time-kill curves were determined for the LVS, Ft6, and Ft24 strains and the leading dcm04 compound. The primary inoculum calibrated at 10^6^ CFU/mL was prepared in MH2-PVX medium and split into five 5-mL aliquots: one drug-free control; three others receiving 4, 8, and 16 times, respectively, the MIC of dcm04 for the tested strain, with 0.5% final concentration of DMSO in all three aliquots; and the last one receiving eight times the MIC of gentamicin for the tested strain, used as a positive control. Sterile MH2-PVX medium with 0.5% DMSO served as a negative control. Cultures were incubated 48 h at 37°C in 5% CO_2_. At 0, 6, 12, 24, 36, and 48 h of incubation, a 50-μL aliquot was taken from each culture after shaking. Then 50 μL of ten-fold serial dilutions of each aliquot was plated on CHA-PVX medium. CFU counts were determined after 72 h incubation of the plates at 37°C in 5% CO_2_. The detection limit was 20 CFU/mL. A 3-log_10_ or more reduction of the primary bacterial inoculum at any incubation time was considered a significant bactericidal effect. Experiments were conducted at least twice to confirm the results.

### Statistical analysis

A statistically significant decrease of viable bacterial counts in MBC assays was evaluated by one-tailed Student t-test using Statview® software. For each antibiotic concentration tested, we compared the bacterial count obtained after 48 h of incubation of cultures to the primary inoculum [i.e., log(N/N0)] and to a 3-log reduction cutoff. Significance was defined as a *p*-value < 0.05.

## Results

### All tested synthetic bis-indolic compounds are active against *F. tularensis* subsp. *holarctica* but not *F. tularensis* subsp. *novicida*

MICs are represented in Tables 2, 3. Almost all *F. tularensis* strains tested were susceptible to the four bis-indole derivatives. In contrast, the Ft5 strain was susceptible to dcm02 and dcm04 (MIC = 8 and 2 μg/mL, respectively), but resistant to dcm01 and dcm03 (MICs > 32 μg/mL). Dcm04 was the most active bis-indole compound with MICs ranging from 2 to 4 μg/mL and a MIC_90_ of 2 μg/mL. The MIC_90_ of the three other compounds was 8 mg/L. It should be noted that prolonged incubation of cultures only increased MICs by one dilution for some compounds. In comparison, the MIC of gentamicin against the LVS, Ft6 and Ft24 strains was 0.25 μg/mL and the MIC of doxycycline was 0.125 μg/mL against LVS and 0.25 μg/mL against the Ft6 and Ft24 strains. As for control strains, the *F. tularensis* subsp. *holartica* LVS strain was susceptible to the four bis-indole derivatives with a MIC of 1–2 μg/mL. In contrast, the reference *F. tularensis* subsp. *novicida* strain and the reference *F. philomiragia* strain were more resistant to these compounds. MICs of all bis-indole derivatives were ≥32 μg/mL for *F. tularensis* subsp. *novicida*, whereas dcm02 and dcm04 displayed lower MICs (16 and 4 μg/mL, respectively) against *F. philomiragia*.

**Table 2:** **MICs (μg/mL) of *Francisella* strains for the four bis-indolic compounds: dcm01, dcm02, dcm03, and dcm04**.

**Bis-indole compound**	***F. tularensis* subsp. *holarctica***				**subsp. *novicida***	***F. philomiragia***
	**FT1 to Ft41**			**LVS**	**CIP 56.12**	**ATCC25015**
	**MIC range**	**MIC_50_**	**MIC_90_**	**MIC**	**MIC**	**MIC**
dcm01	2– >32	4	8	2	>32	>32
dcm02	4– 8	4	8	2	32	16
dcm03	2– >32	4	8	2	>32	>32
dcm04	2– 4	2	2	1	>32	4

**Table 3:** **MICs (μg/mL) and MBCs (μg/mL) of the tree *F. tularensis* subsp. *holarctica* strains used for time-kill studies and MBC determination**.

**Bacterial strain**	**MIC (MBC)**			
	**dcm02**	**dcm04**	**Gentamicin**	**Doxycycline**
*F. tularensis* subsp. *holarctica* Ft6	4	2	0.25 (2)	0.25
*F. tularensis* subsp. *holarctica* Ft24	4	2	0.25 (2)	0.25
*F. tularensis* subsp. *holarctica* LVS	2 (4)	1 (4)	0.25 (2)	0.125

### MBC determination and time-kill studies revealed a bactericidal activity against the LVS strain but only bacteriostatic activity against the Ft6 and Ft24 strains

**Figure 2:**
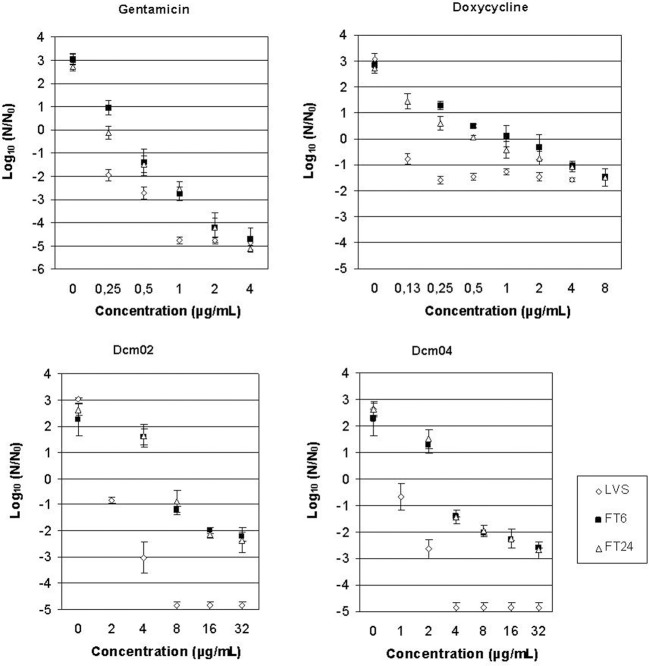
**MBC determination figures for dcm02, dcm04, gentamicin, and doxycycline against *F. tularensis* subsp. *holarctica* LVS, Ft6, and Ft24 strains, performed in triplicate experiments**. Bacterial survival was monitored by measuring CFU/mL after 48 h of incubation. Error bars represent standard deviation.

**Figure 3:**
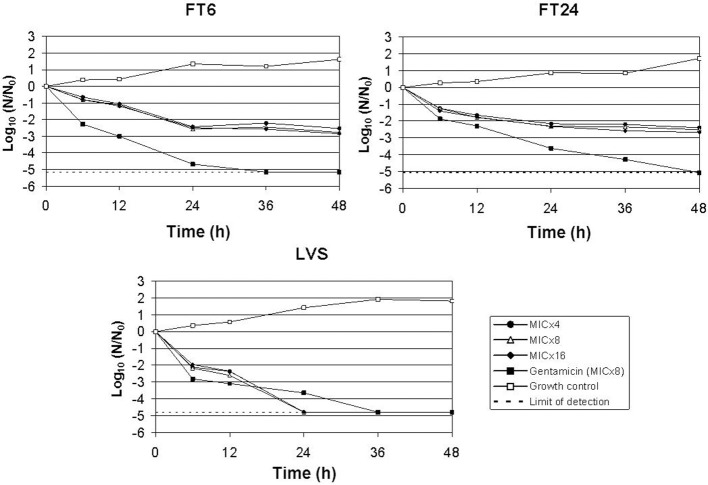
**Time-kill kinetics of dcm04 at 4, 8, and 16 times the MIC against *F. tularensis* subsp. *holarctica* LVS, Ft6, and Ft24 strains**. Gentamicin at eight times the MIC was used as control of a bactericidal antibiotic. Two independent assays were performed but only one representative experiment is shown.

The bactericidal activities of the bis-indole compounds, gentamicin and doxycycline, were determined for the LVS, Ft6, and Ft24 strains (Figures 2, 3). As expected, gentamicin displayed bactericidal activity against the three strains (> 3 log_10_ reduction of the primary bacterial inoculum, *p* < 0.01), with MBCs of 1 μg/mL for the LVS strain and 2 μg/mL for Ft6 and Ft24 strains. As for doxycycline, a significant reduction of the primary bacterial inoculum was observed (i.e., between 1 and 2 log_10_ at MIC × 64 for the LVS strain and MIC × 32 for the Ft6 and Ft24 strains; *p* < 0.01) but the 3-log reduction cutoff was not reached.

MBCs of the dcm02 and dcm04 compounds (4 μg/mL for both compounds) were only two or four times their respective MICs for the LVS strain (Table 3), respectively. In contrast, MBCs could not be determined for the dcm02 and dcm04 compounds against the Ft6 and Ft24 strains. Here again reduction of the primary bacterial inoculum was significant (2-log reduction at concentrations up to MIC × 8 for dcm02 and MIC × 16 for dcm04, *p* < 0.01) but did not reached the 3-log cutoff. Higher concentrations of these compounds could not be tested because of their poor solubility.

Time-kill studies revealed a 3-log_10_ reduction of the primary inoculum of the LVS strain after 12 h incubation for gentamicin (MIC × 8) and 24 h for dcm04 (MIC × 4). As for dcm04, the same bactericidal kinetics were observed at 4, 8, and 16 times the MIC of this compound for the LVS strain. As for the Ft6 and Ft24 strains, we observed a progressive decrease of the bacterial load over the first 48 h of contact with dcm04 (Figure 3), but a 3-log_10_ reduction of the primary bacterial inoculum was never reached. Thus, the dcm02 and dcm04 compounds were only bacteriostatic against the clinical strains of *F. tularensis* subsp. *holarctica*.

## Discussion

Among the currently developed therapeutic alternatives for tularaemia, two promising original classes of compounds have recently been identified. First, substituted diphenyl ethers have demonstrated potent inhibition of ftuFabI Enoyl-acyl carrier protein reductase (England et al., 2009). This enzyme, absent in human cells, plays a key role in the type II fatty acid biosynthesis and has proved to be a useful target for growth inhibition of various pathogens such as *Mycobacterium tuberculosis*, *S. aureus* and *Plasmodium falciparum* (England et al., 2009; Lu et al., 2009; Hevener et al., 2012; Mehboob et al., 2012; Kingry et al., 2013). The leading compound SBPT04 has an MIC of 0.16 ± 0.06 μg/mL against *F. tularensis* LVS and Schu4 strains and also has a bactericidal activity with a MBC of 0.25 μg/mL. In a murine model of *F. tularensis* infection, this compound cleared bacteria by day 4 of treatment, without any relapse the following 30 days post-treatment (England et al., 2009). Secondly, screening of a library of more than 1000 2,5,6- and 2,5,7-trisubstituted benzimidazoles identified 21 leading derivatives exhibiting MICs between 0.35 and 48.6 μg/mL against the *F. tularensis* LVS strain. Their bacterial target remains uncharacterized, but these compounds may block polymerization of FtsZ, which is a homolog of tubulin/microtubule proteins found in eukaryotes, thus interfering with cell division processes (Kumar et al., 2013).

Here, we report that bis-indole derivatives in which the two indole groups are linked either with an amide (dcm01 and dcm02) or an α-keto-amide (dcm03 and dcm04) central linker exhibit antimicrobial activity against *F. tularensis* subsp. *holarctica*. These compounds were previously characterized as anti-staphylococcal drugs active against methicillin-resistant, vancomycin-intermediate, and fluoroquinolone-resistant *S. aureus* strains (Denis et al., 2013a,b). In this study, the 24 bis-indolic molecules evaluated were inactive against Gram-negative bacteria, including enterobacterial species (*Escherichia coli*, *Klebsiella pneumoniae*, *Serratia marcescens*, and *Enterobacter cloacae*), *Pseudomonas aeruginosa* and *Acinetobacter baumannii*. The MICs of two bis-indolic derivatives were lower (16 μg/mL) against *Haemophilus influenzae*. The cytotoxicity of the four bis-indole compounds we tested against *F. tularensis* strains was previously evaluated using three different cell lines: KB (human mouth carcinoma), MCR5 (human lung fibroblast) and HCT116 (human colon tumor) (Denis et al., 2013a,b). The IC_50_ determined using the HCT116 cell line were 1–5 times higher than the MICs found for *F. tularensis* strains.

We found a significant bacteriostatic activity of these tested bis-indole derivatives against 41 strains of *F. tularensis* subsp. *holarctica* isolated in France. Dcm04 appeared to be the most effective compound with a MIC_90_ of 2 μg/mL. The MICs ranged from 2 to 16 μg/mL when considering all four bis-indole compounds, except for a single strain that displayed higher MICs for the dcm01 and dcm03 compounds. The variations in antibiotic activities between the four compounds (especially between dcm01 and dcm03 vs. dcm02 and dcm04) may be related to differences in chemical structure, especially the presence of a large CH_2_NHBoc chemical group in dcm01 and dcm03, whereas it is replaced by a methyl in dcm02 and dcm04. This large chemical group may limit access of dcm01 and dcm03 to their bacterial target or limit their penetration within bacteria. Surprisingly, the dcm02 and dcm04 compounds displayed a bactericidal activity against the virulence-attenuated LVS strain, but not the Ft6 and Ft24 clinical strains of *F. tularensis* subsp. *holarctica*. This was demonstrated both by MBC determinations and in killing curve experiments. The bactericidal activity of dcm04 against the LVS strain was not concentration-dependent but time-dependent. Hopefully, further structural optimization of these bis-indolic compounds and identification of their bacterial targets may enable us to obtain the same bactericidal activity for clinical strains of *F. tularensis* subsp. *holarctica*.

The activity of the bis-indole compounds also showed *Francisella* species and subspecies specificity, since these compounds were active against *F. tularensis* subsp. *holarctica*, only partially active (dcm02 and dcm04) against *F. philomiragia* and inactive against *F. tularensis* subsp. *novicida*. As a result, *F. tularensis* subsp. *novicida* cannot be used as an experimental model to identify the bacterial targets of these compounds, nor to evaluate the *in vivo* activity of the bis-indoles. Comparison of the complete genomes of *F. tularensis* subsp. *holarctica* LVS, OSU18, and FSC200, and that of *F. tularensis* subsp. *tularensis* Schu S4, previously identified a relatively limited number of specific genetic alterations (Petrosino et al., 2006; Rohmer et al., 2006) in the attenuated LVS strain. This might help in further investigations to search for an antibacterial target of these compounds in *F. tularensis*, or at least to explain the differences observed in susceptibility to the bis-indoles. Working hypotheses may include an increased affinity of the bis-indoles for their bacterial target in the LVS strain, a reduced penetration of the bis-indole in the clinical strains as compared to the LVS strain, partial inactivation of the bis-indoles in the clinical strains but not in the LVS strain, an escape pathway to the action of the bis-indoles in clinical strains but not in the LVS strain, and a lower efflux of these molecules in the LVS strain. Another hypothesis is that the bis-indole compounds are more active against the LVS strain because it belongs to the type B biovar II strains of *F. tularensis*, whereas the 41 clinical strains belong to biovar I of this sub-species. Biovar II strains naturally resistant to erythromycin are found in Central and Eastern Europe, and Asia (Kudelina and Olsufiev, 1980), whereas only the erythromycin-susceptible biovar I strains are found in France. We did not evaluate the activity of the bis-indoles against type A *F. tularensis* strains. Testing the activity of these compounds against a larger panel of *F. tularensis* strains will be needed to assess potential variability in susceptibility among different sub-species and biovars.

In conclusion, we have identified novel synthetic bis-indole compounds active against *F. tularensis* subsp. *holarctica* but not the closely related bacteria *F. tularensis* subsp. *novicida* and *F. philomiragia*. These compounds may be drug candidates for the development of new therapeutic alternatives for tularaemia treatment. Their bacterial targets remain to be characterized.

## Author contributions

Research project design: Yvan Caspar, Max Maurin. Experiments: Yvan Caspar, Vivien Sutera, Sandrine Boisset. Writing: Yvan Caspar, Max Maurin.

### Conflict of interest statement

The authors declare that the research was conducted in the absence of any commercial or financial relationships that could be construed as a potential conflict of interest.
